# Protocol for the Emory University African American Vaginal, Oral, and Gut Microbiome in Pregnancy Cohort Study

**DOI:** 10.1186/s12884-017-1357-x

**Published:** 2017-06-01

**Authors:** Elizabeth J. Corwin, Carol J. Hogue, Bradley Pearce, Cherie C. Hill, Timothy D. Read, Jennifer Mulle, Anne L. Dunlop

**Affiliations:** 1 Emory University School of Nursing, 1520 Clifton Rd, Atlanta, GA 30322 USA; 2 Emory University Rollins School of Public Health, Atlanta, GA USA; 3 Department of Gynecology and Obstetrics, Emory University, Atlanta, GA USA; 4 Emory University School of Medicine, Atlanta, GA USA; 5 Emory University School of Nursing and School of Medicine, Atlanta, GA USA

**Keywords:** Microbiome, Chronic stress, Preterm birth, Pregnancy, Health disparity

## Abstract

**Background:**

Adverse birth and neonatal outcomes disproportionately affect African American women and infants compared to those of other races/ethnicities. While significant research has sought to identify underlying factors contributing to these disparities, current understanding remains limited, constraining prevention, early diagnosis, and treatment. With the development of next generation sequencing techniques, the contribution of the vaginal microbiome to adverse maternal and neonatal outcomes has come under consideration. However, most microbiome in pregnancy studies include few African American women, do not consider the potential contribution of non-vaginal microbiome sites, and do not consider the effects of sociodemographic or behavioral factors on the microbiome.

**Methods:**

We conceived our on-going, 5-year longitudinal study, Biobehavioral Determinants of the Microbiome and Preterm Birth in Black Women, as an intra-race study to enable the investigation of risk and protective factors within the disparate group. We aim to recruit over 500 pregnant African American women, enrolling them into the study at 8–14 weeks of pregnancy. Participants will be asked to complete questionnaires and provide oral, vaginal, and gut microbiome samples at enrollment and again at 24–30 weeks. Chart review will be used to identify pregnancy outcomes, infections, treatments, and complications. DNA will be extracted from the microbiome samples and sequencing of the V3 and V4 regions of the 16S rRNA gene will be conducted.

Processing and mapping will be completed with QIIME and operational taxonomic units (OTUs) will be mapped to Greengenes version 13_8. Community state types (CSTs) and diversity measures at each site and time will be identified and considered in light of demographic, psychosocial, clinical, and biobehavioral variables.

**Discussion:**

This rich data set will allow future consideration of risk and protective factors, between and within groups of women, providing the opportunity to uncover the roots of the persistent health disparity experienced by African American families.

## Background

The elimination of disparities in birth and neonatal outcomes experienced by African American women and infants compared to white women and infants in the United States remains a national priority. According to the 2017 National Vital Statistics Reports, [1] African American women face a nearly 50% greater risk of preterm birth (<37 completed weeks of gestation) compared to white women (13.2% vs 8.9%), a difference in risk that doubles (4.8% vs 2.4%) when considering early preterm birth (born < 34 completed weeks’ of gestation). They are also at a higher risk to miscarry [2]. Adverse infant outcomes are likewise unequally distributed, with African American infants more than twice as likely to be born at low (<2500 gm; 13.2% vs 7.0%) or very low (<500 gm; 2.9% vs 1.1%) birthweight [1] and, as from 2013, face a doubling in the risk of total infant mortality (10.81% vs 5.07%). These health disparities are only moderately attenuated by accounting for known socioeconomic and medical risk factors and persist in spite of public health initiatives to improve access to, and the content of care. Faced with this continuing challenge, clinical and scientific attention has increasingly focused on gaining a better understanding of the underlying biobehavioral mechanisms by which the multiple, complex, and often inter-related social, biological and behavioral risk factors disproportionately experienced by African American families link to adverse birth outcomes including preterm birth. Among recognized risk factors for adverse birth outcomes are sociodemographic factors, chronic stress including racial discrimination, [3] dietary threats including micronutrient deficiency [4] and obesity, [5] sexual or hygiene behaviors that heighten the risk of infection or inflammation, [6] and exposure to tobacco or other toxins.

The recent ability to use genomic technologies has led to the knowledge that humans and other vertebrates are colonized by trillions of bacteria, called the microbiome, that directly or indirectly influence health and disease across the lifespan, [7] raising the possibility that these microorganisms may also influence maternal and infant health outcomes. This possibility is especially compelling given that microbiome functions associate with one or more of the known risk factors for adverse pregnancy outcomes including programming and maintenance of the immune system and protection against infection, [8, 9] the physical and emotional response to acute and chronic stress, [10] harvesting of micronutrients and the digestion and metabolism of food, [11] and the breakdown and absorption of toxins [12].

Support for a potential role of the microbiome in influencing birth outcomes comes from recent reports showing that the dominant vaginal microbiome communities present in non-pregnant African American women differ significantly from those present in white women [13, 14]. This raises the possibility that if vaginal microbiome differences persist during pregnancy, a clear link to disparate outcomes might also exist. To date this hypothesis has been difficult to confirm due to the generally small sample size of African American women in most studies of the vaginal microbiome during pregnancy [15–19] and, with few exceptions, [14] the very low representation of African American women delivering infants at term. As a result, the “healthy” vaginal microbiome of pregnant African American women remains poorly characterized. Moreover, no studies to date of the vaginal microbiome of pregnant African American compared to pregnant white women have considered the key biobehavioral variables identified above that may underlie microbial community differences: exposure to chronic or acute stress, diet quality and obesity, concurrent local or systemic infection, reproductive tract hygiene, sexual risk and protective factors, or the use of tobacco or other substances.

Our understanding of the contribution of the microbiome to health disparities in pregnancy outcomes is also limited by the fact that nearly all studies to date have focused only on the vaginal microbiome. This is not unexpected given the potential for microorganisms colonizing the vagina to ascend into the uterine cavity and the recognition that the most common cause of spontaneous preterm birth is infection [6]. Nevertheless, the possibility of cross-contamination of bacteria from the oral or gut environments to the vagina, uterus or placenta, either through hematogenous spread or from direct colonization via oral, vaginal, or anal intercourse, or for the gut microbiome, via cross-contamination after defecation, emphasizes the need for further investigation. To date, however, far fewer studies have investigated other inhabited environments; only one [17] study to date has reported on the oral microbiome using RNA sequencing rather than culture-based methods during gestation, and only three on the gut microbiome in pregnancy, [17, 20, 21] with outcomes from these latter studies conflicting.

The aims of this study are to: 1) Characterize the structure and dynamics of the vaginal, oral, and gut microbiome of African American women in early and later pregnancy by 16S rRNA gene sequencing; 2) Identify biobehavioral factors (including stress, hygiene, and risk and protective behaviors) that influence the structure and dynamics of the vaginal, oral, and gut microbiome; and 3) Evaluate whether the composition of the vaginal, oral, and gut microbiome in early and/or later pregnancy is associated with birth outcomes including gestational age of the infant at birth. The aims of this manuscript are to present the research design, data collection and laboratory methods involved. Importantly, only African American women, the population of pregnant women most under-represented in studies of the microbiome and at highest risk for adverse birth outcomes, will be included. This restriction exists in light of health disparity research recommendations that emphasize that in order to understand and eliminate health disparities, researchers must first look *within the disparity group* to identify the within race norms and the risk and protective factors experienced by the population [22].

## Methods/design

### Study design

The Emory University African American Microbiome in Pregnancy Cohort Study is a prospective cohort study investigating the role of the oral, vaginal, and gut microbiome on pregnancy outcomes, as well as biobehavioral factors that shape the microbiome, among a socio-demographically diverse group of African American women receiving prenatal care in metropolitan Atlanta, Georgia. The aim is to recruit at least 540 women. The study was approved by the Emory University Internal Review Board (IRB) and the appropriate review councils for each hospital.

### Conceptual framework

**Fig. 1:**
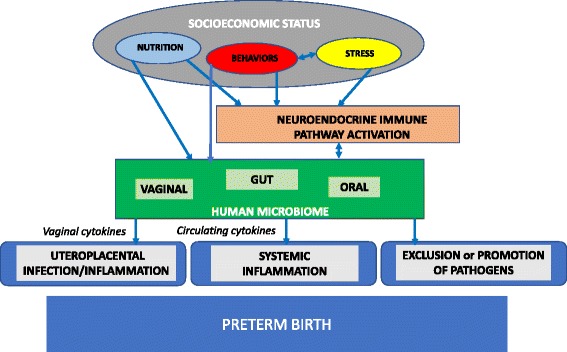
Theoretical framework

All complex health conditions include in their underlying etiology a web of overlapping social, biological and behavioral factors. To date, however, no research has dissected these complex and overlapping associations to consider how they may contribute to inter-individual susceptibility to adverse maternal and infant outcomes in the population at highest risk: African American families. This study is guided by a conceptual framework that posits: stress, nutrition, and health behaviors (operative within the socioeconomic context of AA women’s lives), impact the vaginal, oral, and gut microbiome directly and via neuroendocrine immune pathway activation. The microbiome, in turn, influences the local and systemic inflammatory milieu and excludes or promotes pathogens to ultimately influence birth outcomes (Fig. 1). In this manuscript, we provide the design and data collection details related to our cohort that will ultimately be used to expose the complex mechanisms underlying the health outcomes under investigation.

### Study population and sites

Pregnant women who self-identify as African American (for purposes of this study defined as US-born women of African American or Black race) are recruited to participate in this study from the prenatal care clinics of two metropolitan hospitals in Atlanta, GA, affiliated with Emory University Woodruff Health Sciences Center: Grady Memorial Hospital, a county-supported hospital that serves as a safety net for low-income patients; and Emory University Hospital Midtown, a private hospital that serves patients from a wide economic range. Given that sociodemographic status is a determinant of the stress and health behaviors under study, we anticipate that the diversity across these hospitals will provide sufficient variation in the socio-demographic and biobehavioral factors of interest to probe their risk and protective effects on the microbiome and birth outcomes including preterm birth.

### Participant recruitment

Women who present to either hospital’s prenatal clinic for a first prenatal visit between 8 and 14 weeks gestation, as determined by standard criteria based upon last menstrual period and/or first trimester ultrasound, and self-identify as African American, are provided information about the study by a member of the clinic staff at registration; if expressing interest, they are introduced to an on-site research coordinator and provided a detailed description of the study, including inclusion and exclusion criteria. Subjects meeting criteria who wish to enroll are then asked to provide Informed Consent.

### Inclusion/exclusion criteria

Inclusion criteria are that each participant is: 1) African American by self-report. 2) Between 8 and 14 weeks’ gestation (verified by clinical record and/or ultrasound) and expecting a singleton pregnancy. 3) Able to comprehend written and spoken English. 4) Between 18 and 40 years of age. 5) Experiencing no chronic medical condition or taking prescribed chronic medications (verified by prenatal record), as these may impact risk for adverse birth outcomes. Subjects also are required to live within a 20-mile radius of the laboratory, to minimize the time biological samples are in transport, thereby preserving the validity of the biomarkers examined.

### Data collection

Data collection occurs at three time points during the study: 1) biological samples, and clinical and questionnaire data are collected two times during pregnancy (at the first prenatal care visit occurring between 8 and 14 and at a later prenatal care visit occurring between 24 and 30 weeks); and (2) clinical data (from the medical record) are collected post-delivery. Biological data collected at both prenatal visits include: blood samples for measurement of serum cytokines; self-collected vaginal, oral, and rectal swab samples for microbiome analyses; self-collected vaginal swabs for pH, Gram stain for Nugent’s criteria, and vaginal cytokines. Additional biological data collected at only the first prenatal visit include a blood sample for measurement of dexamethasone suppression (Dex IC_50_) to provide an objective measure of chronic stress. Participants also complete a demographic survey and a battery of measures related to acute and chronic stressors, symptoms of depression and anxiety, substance use, and oral, vaginal, and gut hygiene and sexual practices at both visits. Subjects are compensated at both times. Medical record abstraction is conducted by trained research staff for evidence of pregnancy infections, complications and treatments, and laboratory outcomes. Post-delivery medical record abstraction is detailed for birth outcome (gestational age, birth weight, size-for-age), delivery type, and complications.

### Socio-demographic and psychosocial measures

The following socio-demographic and psychosocial measures are completed at the identified visit(s).

**1. Socio-demographic Survey** (8–14 weeks) is completed using self-report and prenatal administrative record review to gather information on age, years of education, marital status, and insurance status. Family size and household income data are collected and used for determination of poverty/income ratio (PIR).

**2. Health Survey** (8–14 and 24–30 weeks) is completed by self-report to ascertain, within the last month, diagnoses (including infections), medications (including antibiotics), sexual encounters (type of intercourse, use of condoms, number of partners), hygiene self-care practices (douching, feminine sprays/wipes; tooth brushing, flossing, oral rinses), and substance use (tobacco, alcohol, drugs). For each item for which there is an occurrence within the month, the Timeline Followback (TLFB) Interview [23] is used to ascertain the timing of occurrence(s) of the item, with specific probing of occurrence(s) within the 48-h preceding sampling. The TLFB is a calendar-based method that aids participant event recall, and has been validated in low-income and minority populations. Collected data is used to categorize women, for example, as smokers or non-smokers, and will be considered in future analyses to better understand how exposures (including within 48-h, 1-week, and 1-month of sample collection) might impact the microbiome and birth outcomes.

**3. Measures of Psychosocial Stress**: Consistent with the life-course perspective, a range of instruments validated in minority reproductive age women are employed to measure stressors early in life and at present. All instruments are completed at 10–14 weeks and items g-h are repeated at 24–30 weeks. Measures include the following **(a)** ***Childhood Trauma Questionnaire*** (CTQ), which elicits information about abuse and neglect in childhood by asking about 21 experiences and subjects rating their occurrence on a 5-point Likert scale [24]. The CTQ has been extensively used in high-risk populations of African Americans and in a prospective study of pregnant women [25] **(b)** ***Adverse Child Experiences Questionnaire*** (ACE) ascertains experiences related to dysfunction in childhood home by asking about 10 experiences with each being scored 0–1 [26]. ACE is used extensively among women of reproductive age of various race/ethnicity and income status; elevated scores correlate with increased risk for adverse outcomes. **(c)** ***Stressful Live Events Index*** ascertains whether during the past 12 months the woman experienced any of 13 stressful events (e.g., death of a family member, divorce, loss of job, intimate partner violence) with each being scored 0–1, with total scores ranging from 0 to 13. **(d)** ***Krieger Experiences of Discrimination scale*** measures perceptions of usual exposure and response to perceived discrimination in school, home, work and other domains using a Likert-scaled approach [27] **(e)** ***Brief Jackson-Hogue Stress Scale*** is a 36-item scale, with each item Likert scaled 0–5, developed based upon research at Grady Memorial Hospital to capture usual experiences of gendered racism and stress among African American women, [28] including during pregnancy. ***(f) Perceived Stress Survey*** (PSS) is a 14-item questionnaire that measures experiences of stress in the last month [29]. PSS scores have been reported to correlate with HPA axis function during pregnancy and postpartum. ***(g) Spielberger State-Trait Anxiety Inventory*** measures current stress and anxiety as well as anger traits using a 20-item inventory (scored 0–1). It has been widely used in perinatal studies and is well-validated in minority and low-literacy populations [30] **(** ***h) Edinburgh Depression Scale*** (EDS) is a common measure used to ascertain symptoms of depression in the previous 7 days. The EDS has 10-items, is easy to administer, and validated for pre- and postnatal use across multiple race/ethnic groups [31]. Validation against clinical interview identifies a specificity of 78%, sensitivity of 86%, and positive predictive value of 73%. Women scoring >10 on the EDS, or displaying signs of psychological distress, are assisted in seeking help and contacting their care providers.

**4. Nutrition. Food Frequency Questionnaire** (FFQ; (8–14 and 24–30 weeks) is used to ascertain dietary and supplement intake over the previous three months of: vitamin D, folate, PUFAs, essential trace elements, and probiotics(ref). Information is collected using a modified Block-Bodnar semi-quantitative FFQ (modified to quantify probiotic intake) developed to address intake of these nutrients by pregnant women and validated in numerous studies of women of various races/ethnicities and low literacy [32]. Data are collected using NutritionQuest online FFQ, linked to Data-on-Demand, which provides nutrient analysis of the entered data.

### Clinical data

The following clinical data are collected at the identified visit(s).

**1. Clinical Data Collection: Maternal Medical Chart Abstraction** (8–14 & 24–30 weeks, and post-delivery) is completed by the research team using a standardized chart abstraction tool to ascertain for the following pre- and perinatal conditions and birth outcomes:

***(a) Pre-pregnancy BMI***, is calculated from measured height at the first prenatal visit and patient report of pre-pregnancy weight and categorized according to accepted definitions (obesity ≥30 kg/m^2^, overweight 25–29.99 kg/m^2^, healthy weight 18.5–24.99 kg/m^2^, and underweight <18.5 kg/m^2^). ***(b) Gestational age at birth***. All participants receive early pregnancy dating by last menstrual period (LMP) and/or early ultrasound, given enrollment criteria. Gestational age at birth is determined from the delivery record, based upon the date of delivery in relation to the estimated date of confinement established during the 8–14 week prenatal visit. ***(c) Reproductive tract infections***. Clinical diagnoses of reproductive tract infections (BV, specific sexually transmitted infections) during pregnancy are ascertained from the prenatal record, including through prenatal clinical laboratory tests results. ***(d) Complications/Type and Mode of Delivery***. Gestational diabetes, preeclampsia/eclampsia, etc., type and mode of delivery are ascertained from record review after delivery and defined according to standard clinical definitions of the American College of Obstetricians and Gynecologists.

### Biological samples

The following biological samples are collected at 8–14 and 24–30 weeks.

**1. Vaginal, Oral, and Rectal Swabs:** Participants are provided verbal and pictorial instructions directing them to obtain (in a private exam room) five self-collected vaginal swabs (two for DNA sequencing and microbiome analysis and one each for Gram stain, pH, and cytokines); and two oral swabs and two rectal swabs (all for DNA sequencing and microbiome analysis) using validated, field-tested methods and protocols consistent with the Human Microbiome Project [33]. Vaginal: Swabs are immediately handed to the research coordinator (waiting outside the room). The 1st and 2nd swabs are Sterile Catch-All™ Sample Collection Swabs (Epicentre Biotechnologies, Madison WI) which are then stored in MoBio bead tubes (MoBio Laboratories, Inc., Carlsbad, CA) and frozen upright on dry ice until transported to the lab, to be stored at −80 °C until DNA extraction and preparation for vaginal microbiome measurement occurs. The 3rd swab is applied to pH-strips (Merck, Darmstadt, Germany); scoring is completed according to the manufacturer instructions using a scale from 4.0–7.7. The 4th swab is stored frozen at −80 °C for later determination of vaginal cytokines. The 5th swab is rolled onto a microscope glass slide for transport to the Emory Clinical Microbiology laboratory for Nugent criteria scoring for evaluation of BV. Well-designed studies support that vaginal self-collection swabs sample the same microbial diversity as physician-collected swabs of the mid-vagina and have high overall morphotype-specific validity compared with provider-collected swabs based on microbiome analysis [34].

Oral: The oral swabs are stored in MoBio bead tubes (MoBio Laboratories, Inc., Carlsbad, CA) and frozen upright on dry ice until transported to the laboratory where they are stored at −80 °C until DNA extraction and preparation for oral microbiome measurement. Numerous studies support the self-collection of oral mucosal samples.

Rectal: The rectal swabs are stored in MoBio bead tubes (MoBio Laboratories, Inc., Carlsbad, CA) and frozen upright on dry ice until transported to the lab for storage until DNA extraction. Prior to extraction, fecal material (200 mg) is suspended in 500-μl lysozyme (20 mg/ml in 20 mM Tris-HCl pH 8, 2 mM EDTA, 1.2% *w*/*v* Triton X-100) and incubated at 37 °C for 2 h using the QIAamp® DNA Stool Mini Kit (Qiagen, Inc., Valencia, CA). Studies of pregnant women (35–37 weeks) report that self-collection of vaginal-rectal swabs for Group B streptococcus are equivalent to provider-collected swabs and require only simple patient education.

**2. Blood Samples:** For blood sample collections, the research coordinator accompanies the participant to the blood draw station within the prenatal care clinic, for her routine prenatal blood draw at both the 8–14 week and 24–30 week appointments; the laboratory technician, using the same needle stick for the routine blood draws, obtains additional tubes of blood for research purposes to include: At the 8–14 week visit: an additional 30 mL, half collected into serum tubes that are placed on ice for measurement of serum cytokines and aliquoting and storage for future nutrient assays; and half into heparin-containing tubes that remain at room temperature for in vitro DEX suppression (DexIC_50_) testing. At the 24–30 week visit: 12 mL are drawn into serum tubes again for placement on ice for measurement of serum cytokines and aliquoting and storage for future nutrient assays.

### Sample processing and bioassays

**1. DNA Extraction and 16S rRNA Gene Sequencing:** DNA is extracted from swab samples using the MoBio isolation Kit in line with the HMP Standard Operating Protocol [35]. The content of the microbiome is characterized by PCR and sequencing of the V3-V4 region of the 16S rRNA gene, according to well-established protocols.

**2. Vaginal Gram Stain:** At the Clinical Microbiology Laboratories of the delivery hospitals, smeared slides are dried and heat-fixed prior then Gram stained. The slide is then scored by the lab using Nugent’s criteria [36]. Scores of 0–3 are categorized as low, those with 4–6 as intermediate, and those with 7–10 as high scores.

**3. Vaginal and Serum Cytokines:** Vaginal swabs and serum samples are analyzed for cytokines, IL-1β, IL-6, IL-8, TNF-α, and IL-10, using the MesoScale assay platform (Meso Scale Diagnostics Rockville, Maryland) according to the protocols supplied by the manufacturer. The MesoScale multiplex assay system uses electrochemiluminescence for high sensitivity and broad dynamic range.

**4**. **White Blood Cell (WBC) DEX sensitivity:** The procedure for whole blood DEX testing was adapted from previous work [37]. Briefly, 200 μl of whole blood diluted 10:1 with sterile saline is incubated with lipopolysaccharide (LPS; Difco, Augsburg, Germany; final concentration 30 ng/ml) along with increasing concentrations of DEX (Sigma, Deisenhofen, Germany; 10–9 to 10–5 M) in 96 well cell culture plates for 6 h at 37 °C in 5% CO_2_. Culture plates are centrifuged at 2000 X g for 10 min, and supernatants aliquoted into 1 ml polystyrene tubes and transported on ice for storage at −80 °C until assayed for TNF-α, the indicator cytokine (Fisher Scientific, BD Cell Analysis, Atlanta, GA). Immune cell subsets are enumerated using complete blood count with differential (CBC w/diff). The DEX concentration required to suppress 50% of the LPS-induced TNF-α response is determined.

### Data management

Questionnaire and clinical data are directly entered into computer tablets via REDCap management software by research coordinators. Laboratory assay data are imported into the same REDCap database, with the exception of the 16S rRNA gene sequencing data, which is stored in a separate, linked database for ready access and analyses.

### Statistical plan

#### Power and sample size calculations

Sample size calculations were based upon the primary aim to test for differences in microbiome characteristics between groups of women with early births (including preterm and early term births) versus full term births. Given the expected rates of preterm (< 37 weeks; 15%), early term (37–0/7 through 38–6/7; 20%) and full term (≥ 39 weeks; 65%) births in our cohort, our proposed sample size of 540 women will yield a group size of 190 cases of preterm and early term births and an estimated 350 cases of full term birth to comprise the comparison group of full term controls. Power analysis indicated that based on a repeated measures design and Geisser-Greenhouse Corrected F Tests, our sample will have >80% power to detect a difference of 0.25 standard deviation (SD) between-group (an effect size of 0.3) and a difference of 0.1 SD within-group (an effect size of 0.2) in microbiome characteristics over time respectively (at α = 0.05). A 15% attrition was accounted for in the analysis.

### Data analysis

Aim 1. To test for variance in the vaginal, oral, and gut microbiome within and across individuals at 8–14 and 24–30 weeks’ we will make three comparisons within the same woman over time and across individuals over time: (1) the percentage of particular types of predominant microbes and known bacterial pathogens; (2) the multinomial distribution of the various microbial types; (3) the Shannon species diversity index. Two-sample t-test or ANOVA will be used to compare the percentage of particular types of microbes between subject subgroups. To compare the percentage of particular types of microbes within the same women over time, paired t-test will be performed. A multivariate analysis will be used to compare the distributions within the same woman over time. Two-sample t-test/ANOVA or their nonparametric counterparts Wilcoxon Rank-Sum test/Kruskal-Wallis test will be used to compare the Shannon species diversity index between subject subgroups. Paired t-test or Wilcoxon Signed Rank test will be used to compare the Shannon species diversity index within the same woman over time.

Aim 2. To test for the association between and among the biobehavioral factors under study and the structure and dynamics of the vaginal, oral, and gut microbiome (in terms of the particular microbes present, the particular patterns/clusters of microbial types, and the Shannon species diversity index): For bivariate association analyses, we will test the association between the biobehavioral factors and the structure of microbiome using the t-test/ANOVA, Chi-square test and correlation analyses. For multivariate analyses, we will model the percentage with particular microbes at early and later pregnancy using the generalized linear mixed model, model the construction of the microbial types (the percentage distribution of types) at early and later pregnancy using the repeated measures multinomial logit model, and model the Shannon species diversity index at early and later pregnancy using the linear mixed model. We will also control for potential confounding factors. Hypotheses tests based on fitted models will allow us to test whether biobehavioral factors have significant association with the structure of microbiome at early and/or later pregnancy.

Aim 3. To test whether the composition of the vaginal, oral, and gut microbiome at 8–14 and 24–30 weeks is associated with the occurrence of preterm, early term, or full term birth considered as dichotomized categorical variables, and whether specific biobehavioral factors interact with the microbiome composition to influence the risk of early term or preterm birth: For bivariate association analyses, we will perform two sample t-test/Wilcoxon Rank-Sum test to assess whether the percentage with a particular type of predominant microbes and the Shannon species diversity index of the vaginal, oral, and gut microbiome are associated with the occurrence of early term or preterm birth. For multivariable analyses, we will use logistic regression to model the occurrence of early term or preterm birth in relation to the composition of the vaginal, oral, and gut microbiome at 8–14 weeks and 24–30 weeks, while controlling for potential confounding risk factors. We will test the significance of the interaction terms between specific biobehvarioral factors and microbiome compositions in the model to assess whether biobehvarioral factors interact with the microbiome to affect birth outcomes.

## Discussion

In this paper, we present the design, data collection methods, and expected statistical plan for the women participating in the on-going Emory University African American Microbiome in Pregnancy Cohort Study. The women receiving care at the two hospitals from which the women are recruited are different from each other in many ways, and as such are expected to provide a rich depiction of the risk and protective factors experienced by the cohort as a whole. This combination of risks and protective factors, which will include the associated inflammatory and microbiome data for over 500 women, will provide a unique opportunity to look within a disparity population to identify the complex web of interactions that separate healthy from adverse outcomes.

Strengths of the study include the ability to gather high quality clinical (prenatal, labor and delivery) data from the medical record from both hospitals, thereby reducing variability in missing data and imprecision in variables such as gestational age as well as maternal weight and body mass index classification. Additionally, the range of income and education of the women enrolled in the cohort is expected to provide the ability to contrast and compare across and within the recruiting sites and to tease apart the influences of socioeconomic status and various health behaviors both within and across cohorts. Finally, including in the study both psychosocial and biological data collected within the same hour, reduces the possibility that intervening factors would interfere with the ability to accurately identify associations.

No study is without weaknesses. Although chart review will be used to provide objective information whenever possible on risk and protective factors and clinical outcomes, self-report will be the source for important data as well; educational attainment, discrimination, smoking, hygiene etc. However, because of our interest in stress and behavior, a woman’s perceptions of her standing and experiences are significant.

This study’s impact will extend beyond just exposing risk and protective factors influencing birth outcomes. The depth and extent of the data collected from the women participants will stimulate the identification of other important research questions of interest to be asked as well. For example, applying new approaches to the analysis of stored blood samples could provide information on previously unconsidered biomarkers or allow for future identification of mechanistic pathways. Through additional studies such as these, the Emory University African American Microbiome in Pregnancy Cohort Study will extend its impact and increase its potential to reduce health disparities across the lifespan.

## Abbreviations

AA: African American
CBC w/diff: Complete blood count with differential
CSTs: Community state types
DEX IC_50_: Dexamethasone suppression test
DNA: Deoxyribonucleic acid
IRB: Internal review board
LPS: Lipopolysaccharide
OTUs: Operational taxonomic units
PTB: Preterm birth
rRNA: Ribosomal ribonucleic acid
TLFB: Timeline followback
