# Correction to: Protocol for the Emory University African American Vaginal, oral, and gut microbiome in pregnancy cohort study

**DOI:** 10.1186/s12884-017-1550-y

**Published:** 2017-11-27

**Authors:** Elizabeth J. Corwin, Carol J. Hogue, Bradley Pearce, Cherie C. Hill, Timothy D. Read, Jennifer Mulle, Anne L. Dunlop

**Affiliations:** 1 Emory University School of Nursing, 1520 Clifton Rd, Atlanta, GA 30322 USA; 2 Emory University Rollins School of Public Health, Atlanta, GA USA; 3 Department of Gynecology and Obstetrics, Emory University, Atlanta, GA USA; 4 Emory University School of Medicine, Atlanta, GA USA; 5 Emory University School of Nursing and School of Medicine, Atlanta, GA USA

## Correction

Following publication of the original article [1], the authors pointed out that the Methods included one step that is no longer necessary but which was inadvertently carried over from an earlier protocol.
